# CLDN6 promotes chemoresistance through GSTP1 in human breast cancer

**DOI:** 10.1186/s13046-017-0627-9

**Published:** 2017-11-07

**Authors:** Minlan Yang, Yanru Li, Xiangfeng Shen, Yang Ruan, Yan Lu, Xiangshu Jin, Peiye Song, Yantong Guo, Xiaoli Zhang, Huinan Qu, Yijia Shao, Chengshi Quan

**Affiliations:** The Key Laboratory of Pathobiology, Ministry of Education, College of Basic Medical Sciences, Jilin University, 126 Xinmin Avenue, Changchun, Jilin 310021 People’s Republic of China

**Keywords:** CLDN6, Chemoresistance, GSTP1, p53, Breast cancer

## Abstract

**Background:**

Claudin-6 (CLDN6), a member of CLDN family and a key component of tight junction, has been reported to function as a tumor suppressor in breast cancer. However, whether CLDN6 plays any role in breast cancer chemoresistance remains unclear. In this study, we investigated the role of CLDN6 in the acquisition of chemoresistance in breast cancer cells.

**Methods:**

We manipulated the expression of CLDN6 in MCF-7 and MCF-7/MDR cells with lv-CLDN6 and CLDN6-shRNA and investigated whether CLDN6 manipulation lead to different susceptibilities to several chemotherapeutic agents in these cells. The cytotoxicity of adriamycin (ADM), 5-fluorouracil (5-FU), and cisplatin (DDP) was tested by cck-8 assay. Cell death was determined by DAPI nuclear staining. The enzyme activity of glutanthione S-transferase-p1 (GSTP1) was detected by a GST activity kit. Then lv-GSTP1 and GSTP1-shRNA plasmids were constructed to investigate the potential of GSTP1 in regulating chemoresistance of breast cancer. The TP53-shRNA was adopted to explore the regulation mechanism of GSTP1. Finally, immunohistochemistry was used to explore the relationship between CLDN6 and GSTP1 expression in breast cancer tissues.

**Results:**

Silencing CLDN6 increased the cytotoxicity of ADM, 5-FU, and DDP in MCF-7/MDR cells. Whereas overexpression of CLDN6 in MCF-7, the parental cell line of MCF-7/MDR expressing low level of CLDN6, increased the resistance to the above drugs. GSTP1 was upregulated in CLDN6-overexpressed MCF-7 cells. RNAi –mediated silencing of CLDN6 downregulated both GSTP1 expression and GST enzyme activity in MCF-7/MDR cells. Overexpresssion of GSTP1 in CLDN6 silenced MCF-7/MDR cells restored chemoresistance, whereas silencing GSTP1 reduced the chemoresistance due to ectopic overexpressed of CLDN6 in MCF-7 cells. These observations were also repeated in TNBC cells Hs578t. We further confirmed that CLDN6 interacted with p53 and promoted translocation of p53 from nucleus to cytoplasm, and both the expression and enzyme activity of GSTP1 were regulated by p53. Clinicopathologic analysis revealed that GSTP1 expression was positively associated with CLDN6 in human breast cancer samples.

**Conclusion:**

High expression of CLDN6 confers chemoresistance on breast cancer which is mediated by GSTP1, the activity of which is regulated by p53. Our findings provide a new insight into mechanisms and strategies to overcome chemoresistance in breast cancer.

**Electronic supplementary material:**

The online version of this article (10.1186/s13046-017-0627-9) contains supplementary material, which is available to authorized users.

## Background

Breast cancer is one of the most common cancers in the world. Abnormal expression of claudins (CLDNs) has been interpreted as a mechanism of the malignant progression of cancer [1]. We cloned and identified the claudin-6 (CLDN6) gene, one of 27 members in the CLDN family, low levels of CLDN6 expression was observed in both human and rat mammary cancer [2, 3]. Besides, we have found that CLDN6 function as a tumor suppressor in breast cancer cells, inhibiting the malignant phenotype of breast cancer cells, such as growth, migration, and invasion via p38/MAPK pathway [4]. And we also reported that CLDN6 induced human breast cancer cells apoptosis via ASK1 signaling [5]. These observations indicate that CLDN6 functions as a tumor suppressor in breast cancer. However, we also found that CLDN6 was highly expressed in multidrug resistant breast cancer cell line MCF-7/MDR that derived from human MCF-7 cell line. Besides, studies demonstrated that other CLDNs expression were associated with chemoresistance in cancers [6–8]. We therefore hypothesized that CLDN6 may also play a role in conferring chemoresistance on breast cancer cells.

Resistance to chemotherapeutic drugs is a significant obstacle in the treatment of patients with breast cancer. The acquisition of a multidrug resistant phenotype in metastatic breast cancer is primarily responsible for the failure of current treatment measures. As the development of effective therapies against chemoresistant tumors is a high priority in breast cancer, identification of chemoresistance associated genes is critical for the successful treatment of breast cancer. Chemoresistance correlates with many different biochemical changes, including decreased influx and increased efflux of the cytotoxic drugs as well as altered expression of genes that control cell cycle and apoptosis [9]. Cytostatic drugs can also be detoxified by enzymes, for example glutathione transferases (GSTs) can be induced during chemotherapy. As numerous previous studies have shown that cells selected in vitro for resistance to various types of agents, increased levels of GSTs were often associated with the emergence of resistance to some agents in drug-selected cell lines [10]. The GSTs are multifunctional enzymes that are associated with cellular detoxification, and because they catalyze the conjugation of reduced glutathione to hydrophobic electrophilic compounds, it is believed that GSTs affect mutagenesis and carcinogenesis. Among the 4 GST isozymes, glutanthione S-transferase-p1 (GSTP1) has been reported to play an important role in the resistance of cancer cells to alkylating agents [11]. In ADM resistance breast cancer cell line MCF-7/ADR, upregulation of GSTP1 confers resistance to ADM [12]. In several types of cancers, overexpression of GSTP1 was associated with decreased treatment response and survival. GSTP1 expression was closely associated with the response to chemotherapy and the clinical outcome of breast cancer [13–16]. While it has been reported that there was no significant association between high GSTP1 expression and outcome with improved adjuvant chemotherapy in early breast cancer [10], GSTP1 positive expression showed a significant correlation with a lower histological grade carcinoma in invasive breast cancer [17]. In ER-negative breast cancer, GSTP1 expression predicted a poor pathological response to neoadjuvant chemotherapy [14]. Furthermore, in osteosarcoma and prostate cancer, GSTP1 expression was associated with the chemosensitivity [17, 18]. Whether GSTP1 and CLDNs playing significant roles in different biological characteristics of cancers is not clear, we hypothesize that there may be a close correlation in activity/function between CLDNs and GSTP1, which may provide a possible direction for cancer chemotherapy. Recently a study confirmed CLDN23 as a potential biomarker for the diagnosis and prognosis of gastric cancer, and it was further demonstrated that CLDN23 expression was positively correlated with GSTP1 activity in gastric cancer [19]. Abnormal high CLDN6 expression was found in MCF-7/MDR multidrug resistant breast cancer cells, however whether CLDN6 confers resistance to various anti-cancer drugs in this cell line need to be explored. In the current study, we investigated the effects of CLDN6 expression in response to various anti-cancer drugs in breast cancer, and explore the role of GSTP1 in CLDN6 mediated chemoresistance in human breast cancer cells.

## Methods

### Cell culture and reagents

Human breast cancer cell lines MCF-7, MDAMB231, Hs578t, and human embryonic kidney (HEK) 293T cells were obtained from the Cell Bank of the Chinese Academy of Sciences (Shanghai, China). Human breast cancer multidrug resistance cell MCF-7/MDR was purchased from the Cell Bank of Xiangya Medical College, Central South University. Cells were cultured in Dulbecco’s Modified Eagle’s Medium (Gibco, California, USA) supplemented with 10% fetal bovine serum (FBS) (Thermo Fisher Scientific, Beijing, China), 10 mmol/l HEPES, at 37 °C and 5% CO_2_. Adriamycin (ADM), 5-fluorouracil (5-FU), and cisplatin (DDP) were purchased from sigma (Sigma, MO, USA).

### Plasmid

CLDN6 shRNA plasmid, i.e. pGCsilencer-U6/Neo/GFP was constructed which also expresses Green Fluorescent Protein (GFP) (KeyGEN Biotech, Nanjing, China). Target sequence is GGCAAGGTGTACGACTCA. A functional non-targeting siRNA was used as a control.

A CLDN6 overexpression plasmid was generated by sub-cloning human CLDN6 cDNA (NCBI RefSeq record: NM_021195) into the mammalian expression plasmid EX-LV201 (GeneCopoeia, Guangzhou, China) with FLAGs SV40-eGFP-IRES-puromycin.

Lentivirus production and stable knockdown of the GSTP1 genes shRNA plasmid (sh-GSTP1–21:5′**-**GCCCTACACCGTGGTCTATTT-3′,sh-GSTP1–22:5′**-**AGGACCTCCGCTGCAAATACA-3′ and sh-GSTP1–23:5′-GGCAAGGATGACTATGTGAAG-3′) and negative-shRNA as shRNA-control (sh-GSTP1-CT) were purchased from GeneCopoeia (GeneCopoeia, Guangzhou, China). Silencer negative control with no significant homology to any known human genes was used as a negative control shRNA with FLAGs U6-mCherry-Hygromycin.

Lentivirus production and stable knockdown of the TP53 gene shRNA interference (shRNAi) plasmids were inserted into the psi-LVRU6MH vector downstream of the U6 promoter with FLAGs U6-mCherry-Hygromycin (GeneCopoeia, Guangzhou, China). Plasmids sh-TP53–32 (5′-GGAAATTTGCGTGTGGAGTAT-3′, sh-TP53–33 (5′-CCACTACAACTACATGTGTAA-3′) and sh-TP53–34 (5′-GGACTTCCATTTGCTTTGTCC-3′) were used to knockdown the TP53 gene and a functional non-targeting shRNA (sh-TP53-CT) was served as a negative control clone. For stable RNAi, lentiviral particles were produced.

A GSTP1 overexpression plasmid was generated by subcloning human GSTP1 cDNA (NCBI RefSeq record: NM_000852) into the mammalian expression plasmid EX-LV206 with FLAGs SV40-mCherry-IRES-puromycin (GeneCopoeia, Guangzhou, China).

Lentiviral packaging plasmids expressing gag-pol, rev, and pVSVG genes were obtained from Institute of Biochemistry and Cell Biology of Shanghai Life Science Research Institute, Chinese Academy of Sciences. These vectors were transfected into 293T cells by FuGene HD (Roche Applied Science, Basel, Switzerland). Viral supernatants were harvested at 48 h and 72 h after transfection and concentrated by ultracentrifugation. Viruses were transduced in the presence of 5 μg/mL polybrene.

### Reverse-transcription PCR (RT-PCR) and quantitative RT-PCR (qRT-PCR) analysis

Total RNA was isolated from 5 × 10^6^ cells using TRIzol reagent (Invitrogen, California, USA), according to the manufacturer’s instructions. Total RNA concentration and purity were analyzed in duplicate samples using a Nanodrop ND-2000 spectrophotometer (Thermo Fisher Scientific, MA, USA). cDNA was synthesized from the qualified RNA using an RT-PCR reverse transcription kit (TransGen Biotech, Beijing, China). 1000 ng of total RNA was reverse transcribed into cDNA under the condition: 25 °C 10 min, 42 °C 30 min, and 85 °C 5 s, as manufacturer’s recommendations. Then the cDNA was stored at −20 °C until use. The PCR was performed using a PCR kit (TransGen Biotech, Beijing, China). The PCR product was electrophoresed on 1.5% agarose gels. Primers were synthesized by Sangon (Sangon, Shanghai, China). Quantitative PCR was carried out with either Taq-Man or SYBR Green PCR reagents on an ABI Prism 7300 detection system (all from Applied Biosystems, Foster City, CA). The reaction program was consisted of 95 °C for 3 min followed by 40 cycles of 95 °C 30 s, 55 °C 20 s, and 72 °C 15 s. GAPDH gene was served as internal control and the relative mRNA levels were calculated by 2^−ΔΔCt^. Primers designed were synthesized by Sangon (Shanghai, China).

### Western blot analysis

When cells reach 80% confluence, cells were harvested and washed with PBS. Total protein was extracted with 200 μL RIPA Lysis Buffer (Beyotime, Shanghai, China) with 1 mM phenylmethylsulfonyl fluoride (PMSF) (Sigma, MO, USA) and 1 mM Phosphatase inhibitor (Solarbio, Beijing, China), followed with centrifugation 12,000 *g* for 20 min. The protein concentration was determined using a BCA Protein Assay Kit (Beyotime, Shanghai, China). 50 μg of denatured protein was applied to 12% SDS-PAGE gels. After electrophoresis the proteins on the gel were then transferred onto a nitrocellulose membrane (Millipore, California, USA). The membrane was blocked with 5% defatted milk at 37 °C for 1 h and incubated overnight at 4 °C with the primary antibodies. The membrane was incubated with horseradish peroxidase-conjugated secondary antibodies at room temperature for 1 h. Finally the immunoreactive bands were visualized using an ECL western blotting system (Beyotime, Shanghai, China). The following antibodies were used: a monoclonal mouse anti-β-actin antibody (Santa Cruz Biotechnology, California, USA), a polyclonal rabbit anti-CLDN6 antibody (Santa Cruz Biotechnology, California, USA), a polyclonal rabbit anti-cleaved-caspase-9 antibody (Cell Signaling Technology, MA, USA), a polyclonal rabbit anti-cleaved-PARP antibody (Cell Signaling Technology, MA, USA), a polyclonal rabbit anti-γH2AX antibody (Cell Signaling Technology, MA, USA), a polyclonal rabbit anti-p-Ser15-p53 antibody (Cell Signaling Technology, MA, USA), and a polyclonal mouse anti-GSTP1 antibody (Cell Signaling Technology, MA, USA).

### In vitro drug sensitivity assay

In vitro drug cytotoxicity was measured by Cell Counting Kit-8 (CCK-8) assay (Dojindo, Kumamoto, Japan). The cells were seeded into 96-well plates (3 × 10^3^ cells/well) and then treated for 48 h in 100 μL of medium with anticancer drugs. The cells incubated without drugs (i.e. control wells) were set at 100% survival and were utilized to calculate the concentration of each cytostatic drug lethal to 50% of the cells (IC_50_). CCK-8 reagent was then added and then incubated at 37 °C for 2 h. The optical density (OD) of each well at 450 nm was recorded on a Microplate Reader (Thermo, Schwerte, Germany). The cell viability (% of control) is expressed as the percentage of (OD_test_ − OD_blank_)/(OD_control_ − OD_blank_). The assay was conducted in three replicate wells for each sample and three parallel experiments were performed.

### Apoptosis assay

4′,6-diamidino-2-phenylindole (DAPI) staining was used to detect apoptosis in vitro. Cells were harvested when grown to 60-80% confluency, and treated with ADM for 48 h, then fixed with 4% paraformaldehyde, stained with the 1 mg/mL DAPI (Sigma, MO, USA) for 15 min and examined by fluorescence microscopy to determine the fraction of apoptotic cells. Apoptotic cells were recognized as chromatin condensed, punctate nuclear ghosts with stained, degraded nuclei when examined by fluorescence microscopy. The incidence of apoptosis was analyzed by counting nuclear deep dyeing cells with condensed chromatin, and determining the percentage of apoptotic cells.

### GST activity assay

GST activity was measured using a GST activity kit (Solarbio, Beijing, China) according to the manufacturer’s protocol. It was defined as the amount of enzyme that was required to reflect the ability to reduce GSH and 1-chloro-2, 4-dinitrobenzene (CDNB). The changes in absorbance of the GSH and CDNB were recorded at 340 nm for 10 s and 310 s respectively. GST activity was expressed as nmol per min per mg of total protein concentration.

### Nuclear/cytosol fractionation

To monitor the nuclear and cytosol p53 protein level after CLDN6 overexpression, nuclear/cytosol fractionation along with immunoblotting analysis were performed. 1 × 10^6^ cells were needed. Nuclear/Cytosol Fractionation Kit (TransGen Biotech, Beijing, China) was applied to isolate nucleus and cytosol protein according to the manufacturer’s instructions.

### Immunoprecipitation–western blots

The cells were lysed in IP lysis buffer (Beyotime, Shanghai, China) for 30 min on ice, vortex for 10 s interval of 5 min, then transferred to a 1.5 mL microcentrifuge tube and centrifuged for 20 min at 14,000 *g* to remove cellular debris. The supernatants were analyzed for total protein content, and 300 μg of total protein was incubated with 25 μL of agarose-immobilized goat polyclonal anti-rabbit antibody in a final volume of 500 μL, adjusted with lysis buffer. Immunoprecipitation was carried out with gentle rocking, overnight at 4 °C. The agarose beads were pelleted by centrifugation at 3000 rpm for 5 min, and then washed 3 times with 1 mL lysis buffer, with each wash followed by a 3 min centrifugation at 3000 rpm. After the final wash, 24 μL lysis buffer and 6 μL of 5× SDS sample buffer was added to the beads, the samples were boiled and then loaded onto 12% SDS-PAGE gels. Following protein transfer to PVDF membrane (Millipore, California, USA), p53 and CLDN6 expression were detected by western blotting as described earlier.

### Immunohistochemistry

Immunohistochemistry of tumor tissues collected from human patients breast cancer samples were performed as we described elsewhere [2]. 40 patients with breast cancer at the department of pathology of the second hospital of Jilin university who had not been treated with any chemotherapy and those received neoadjuvant chemotherapy for relapsed disease after initial biopsy either for organ preservation or for unresectable disease. Formalin-fixed, paraffin-embedded biopsy tissues were available. Immunohistochemistry was performed as described elsewhere. Tissue sections were immunostained with CLDN6 antibody (Abcam, MA, USA) and GSTP1 antibody (Cell Signaling Technology, MA, USA). Diaminobenzidine (DAB) was used for color development. CLDN6 expression is indicated in brown and is expressed in the membrane of breast cancer cells and GSTP1 is indicated in brown and expressed in the nuclear of breast cancer cells.

Immunoreactivity was evaluated independently by two observers without specific knowledge of the patients. The staining was graded for intensity (0-negative, 1-weak, 2-moderate, and 3-strong) and percentage of positive cells as follows: 0, <5% positive tumor cells; 1, 5%–25% positive tumor cells; 2, 26%–50% positive tumor cells; 3, 51%–75% positive tumor cells; 4, >75% positive tumor cells. The grades were multiplied to determine an H-score. The H-scores for tumors with multiple scores were averaged. Protein expression was then defined as negative (H-score = 0), weakly positive (H-score = 1–4), moderately positive (H-score = 5–8), and strong (H-score = 9–12). Positive or negative reactions were determined in five random fields of each sample with image processing software Image-Pro Plus 6.0.

### Statistical analysis

All experiments were performed in triplicate and data was reported as means ± SD. Statistical analysis was performed with SPSS 19.0 software. Statistical significance was determined by Student’s *t*-test or one-way analysis. Correlation between CLDN6 and GSTP1 was evaluated using Spearman’s test. *P* < 0.05 was considered statistically significant.

## Results

### Silencing CLDN6 inhibits chemoresistance of MCF-7/MDR cells

**Fig. 1:**
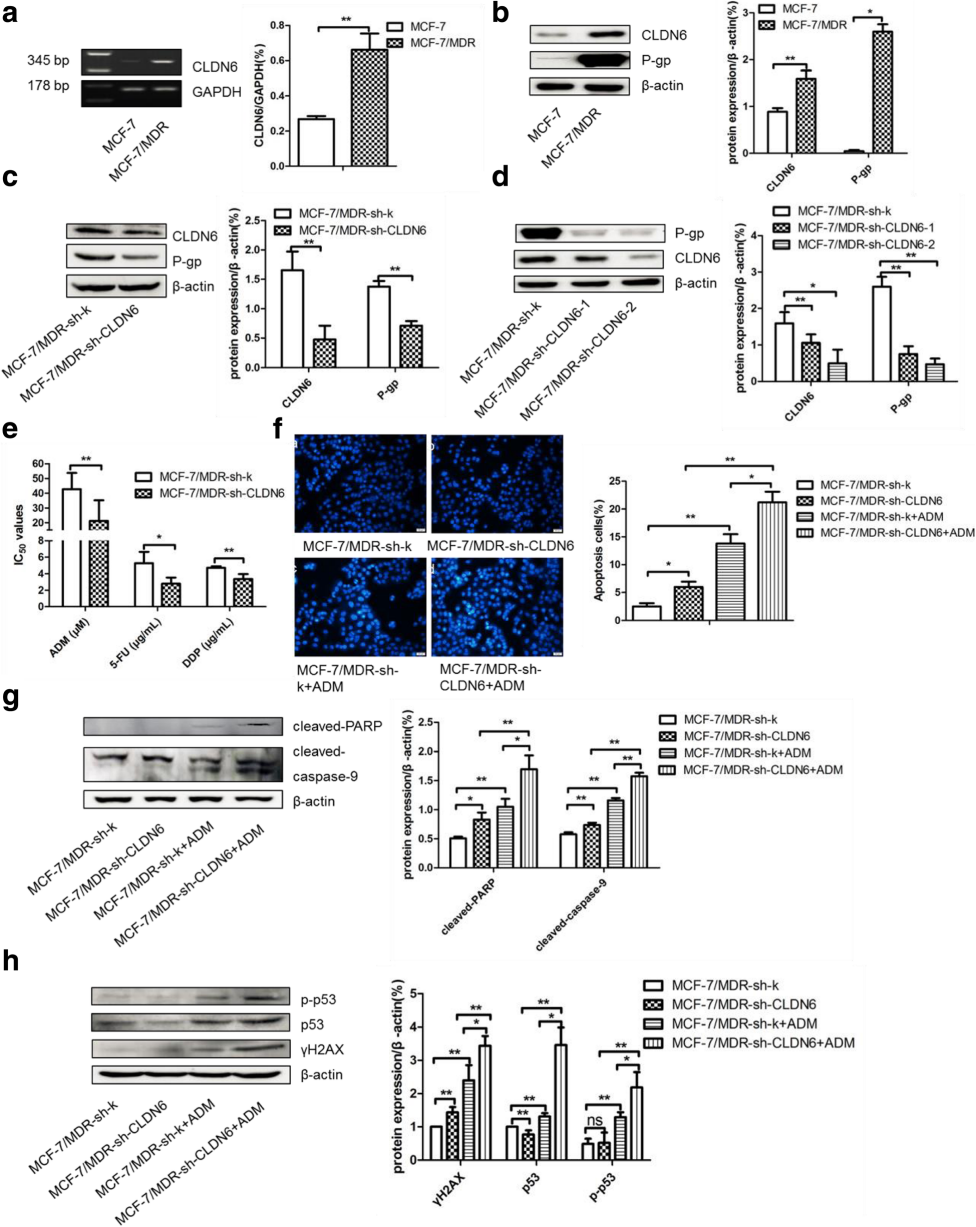
Silencing CLDN6 affected chemoresistance in response to ADM in MCF-7/MDR cells. **a** RT-PCR analysis of CLDN6 expression in MCF-7/MDR and MCF-7 cells. GAPDH was used as a control. **b** CLDN6, P-gp and GSTP1 expression in MCF-7/MDR and MCF-7 cells by using western blot assay. **c** P-gp and CLDN6 expression when silencing CLDN6 transiently in MCF-7/MDR cells by using western blot analysis. **d** P-gp and CLDN6 expression when silencing CLDN6 in screened stable clones in MCF-7/MDR cells by using western blot analysis. **e** IC_50_ of ADM, DDP, and 5-FU when RNAi CLDN6 by shRNA in MCF-7/MDR cells. **f** DAPI nuclear staining was applied to detect ADM-inducing apoptosis in CLDN6-silenced MCF-7/MDR cells (MCF-7/MDR-sh-CLDN6). **g** Cleaved-PARP and cleaved-caspase-9 were detected in MCF-7/MDR-sh-CLDN6 cells by using western blot. **h** Expression of γH2AX, p53, and p-p53 in MCF-7/MDR-sh-CLDN6 cells when treated with ADM. *, *P* < 0.05; **, *P* < 0.01

We previously showed that CLDN6 was significantly downregulated in human breast cancer tissues compared to the adjacent tissues, and CLDN6 expression was relatively low in breast cancer cell lines MCF-7 and MDAMB231. In consistence with large numbers of reports, that expression of P-gp (which is encoded by MDR1) is higher in MCF-7/MDR cells which is resistant to various anticancer drugs. We found that the level of CLDN6 in MCF-7/MDR cells was higher compared to MCF-7 cells (Fig. 1a and b), suggesting that CLDN6 may be a key element in conferring multidrug resistance of breast cancer.

To test whether CLDN6 affects chemoresistance in breast cancer cells, we utilized RNAi to transiently (Fig. 1c) or stalely (Fig. 1d) inhibit CLDN6 expression in MCF-7/MDR cells and we found that expression of P-gp also decreased simultaneously. Parental and RNAi treated MCF-7/MDR cells were challenged with ADM, 5-FU, and DDP in different concentrations for 48 h and cytotoxicity was examined by cck8 assay. As shown in Fig. 1e, silencing CLDN6 resulted in a decrease of IC_50_ of ADM from 42.78 ± 10.95 μM to 21.19 ± 14.02 μM, 5-FU from 5.26 ± 1.39 μg/mL to 2.80 ± 0.75 μg/mL, and DDP from 4.28 ± 0.18 μg/mL to 3.37 ± 0.59 μg/mL, respectively.

When cells treated with 20 μM ADM, apoptosis-associated proteins such as cleaved-PARP and cleaved-caspase-9 (as caspase-3 is lack of expression in MCF-7 cells) were dramatically increased as detected by western blot. DAPI nuclear staining also demonstrated a significant increase of DNA fragmentation in MCF-7/MDR-sh-CLDN6 cells (Fig. 1f) (*P* < 0.05). Moreover, MCF-7/MDR-sh-CLDN6 showed more cleaved-PARP and cleaved-caspase-9 than MCF-7/MDR-sh-k with the treatment of ADM (Fig. 1g), suggesting that ADM induced apoptosis in MCF-7/MDR cells is caspase-9 pathway dependent. We also found a robust induction of apoptosis upon DDP, and silencing CLDN6 showed a synergistic effect (Additional file 1: Figure S1). An increase of γH2AX, a hallmark of DNA damage response, in MCF-7/MDR cells was observed when treated with ADM. Silencing CLDN6 promoted ADM inducing γH2AX generation and phosphorylation of p53 on Ser15 (p-p53) (Fig. 1h). Whereas, silencing CLDN6 had little effect on cell cycle distribution of MCF-7/MDR cells (Additional file 2: Figure S2). The results suggesting that silencing CLDN6 inhibited chemoresistance of MCF-7/MDR cells was correlated with the induction of apoptosis and DNA damage, not due to cell cycle arrest.

### Overexpression of CLDN6 confers chemoresistance on MCF-7 cells

**Fig. 2:**
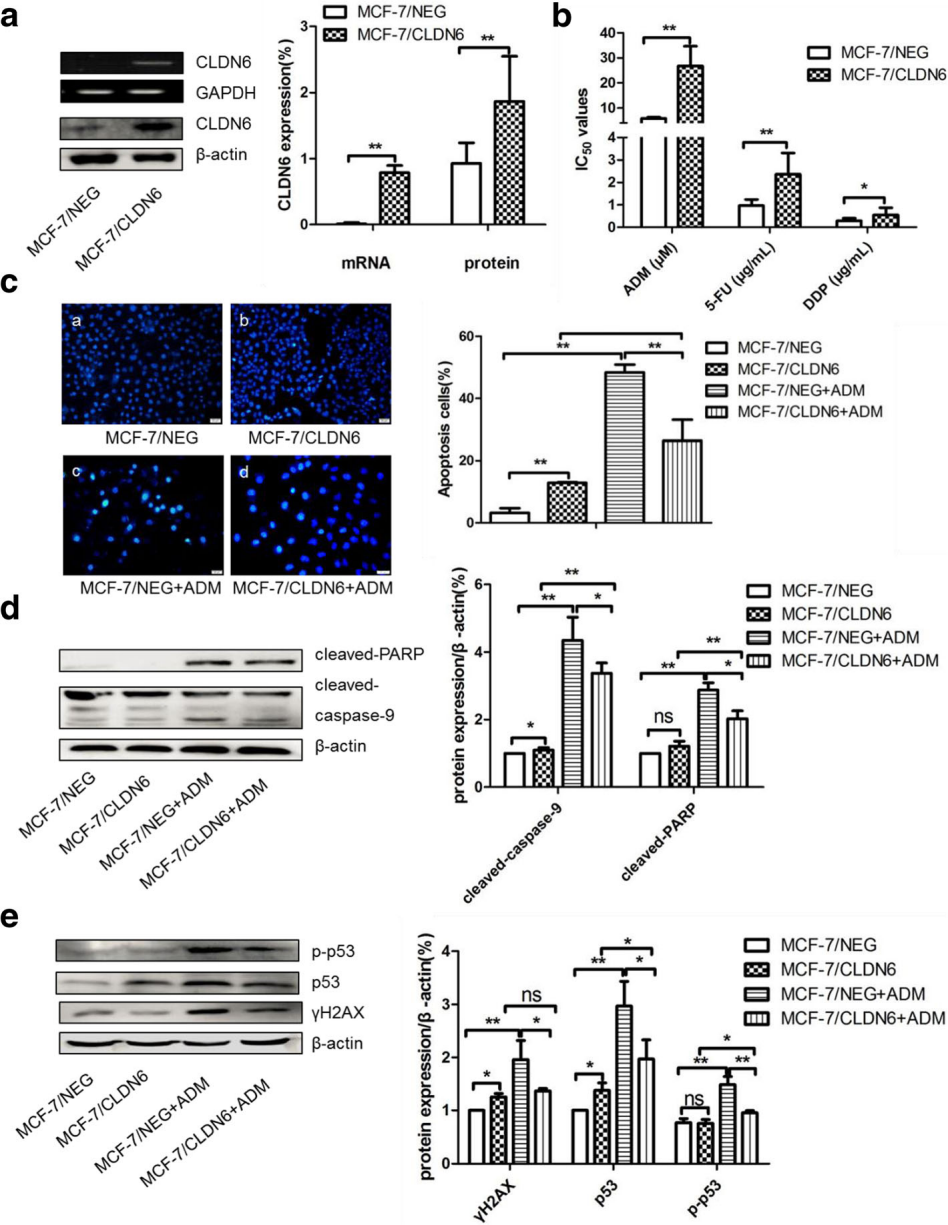
Overexpression of CLDN6 affected chemoresistance in response to ADM in MCF-7 cells. **a** RT-PCR and western blot verified CLDN6 overexpression in CLDN6-overexpressed MCF-7 cells (MCF-7/CLDN6). **b** IC_50_ of ADM, DDP, and 5-FU when overexpressed CLDN6 in MCF-7 cells. **c** DAPI nuclear staining was applied to detect ADM-induced apoptosis in MCF-7/CLDN6 cells. **d** Apoptosis associated proteins expression in MCF-7/CLDN6 treated with the same dose of ADM by using western blot assay. **e** Expression of γH2AX, p53, and p-p53 in MCF-7/CLDN6 cells when treated with ADM. *,*P* < 0.05; **, *P* < 0.01

We then overexpressed CLDN6 in MCF-7 cells to determine whether CLDN6 affects chemoresistance in breast cancer cells. Overexpression of CLDN6 in MCF-7 cells (MCF-7/CLDN6) was verified by RT-PCR and western blot (Fig. 2a). MCF-7/CLDN6 cells were treated with ADM, 5-FU, and DDP at different concentrations for 48 h, and cytotoxicity was examined by the cck8 assay. As shown in Fig. 2b, CLDN6 overexpression dramatically increased the IC_50_ of ADM from 5.81 ± 0.52 μM to 26.74 ± 7.92 μM, 5-FU from 0.96 ± 0.27 μg/mL to 2.36 ± 0.96 μg/mL, and DDP from 0.28 ± 0.11 μg/mL to 0.54 ± 0.32 μg/mL, respectively.

When cells treated with 10 μM ADM, demonstrated a significant decrease of apoptosis by DAPI nuclear staining (*P* < 0.05) (Fig. 2c) and apoptosis proteins cleaved-PARP and cleaved-caspase-9 (Fig. 2d) in the MCF-7/CLDN6 cells. Similarly, when treated with DDP, overexpression of CLDN6 partly reversed DDP induced apoptosis in MCF-7 cells (Additional file 3: Figure S3). Besides, p-p53 and γH2AX induced by ADM were partly inhibited when CLDN6 overexpressed (Fig. 2e). Whereas, overexpression of CLDN6 made no difference in DDP inducing cell cycle arrest (Additional file 4: Figure S4). Consistent with above results, CLDN6 conferring chemoresistance was correlated with the induction of apoptosis and DNA damage in MCF-7 cells.

### CLDN6-conferred chemoresistance is mediated by GSTP1

**Fig. 3:**
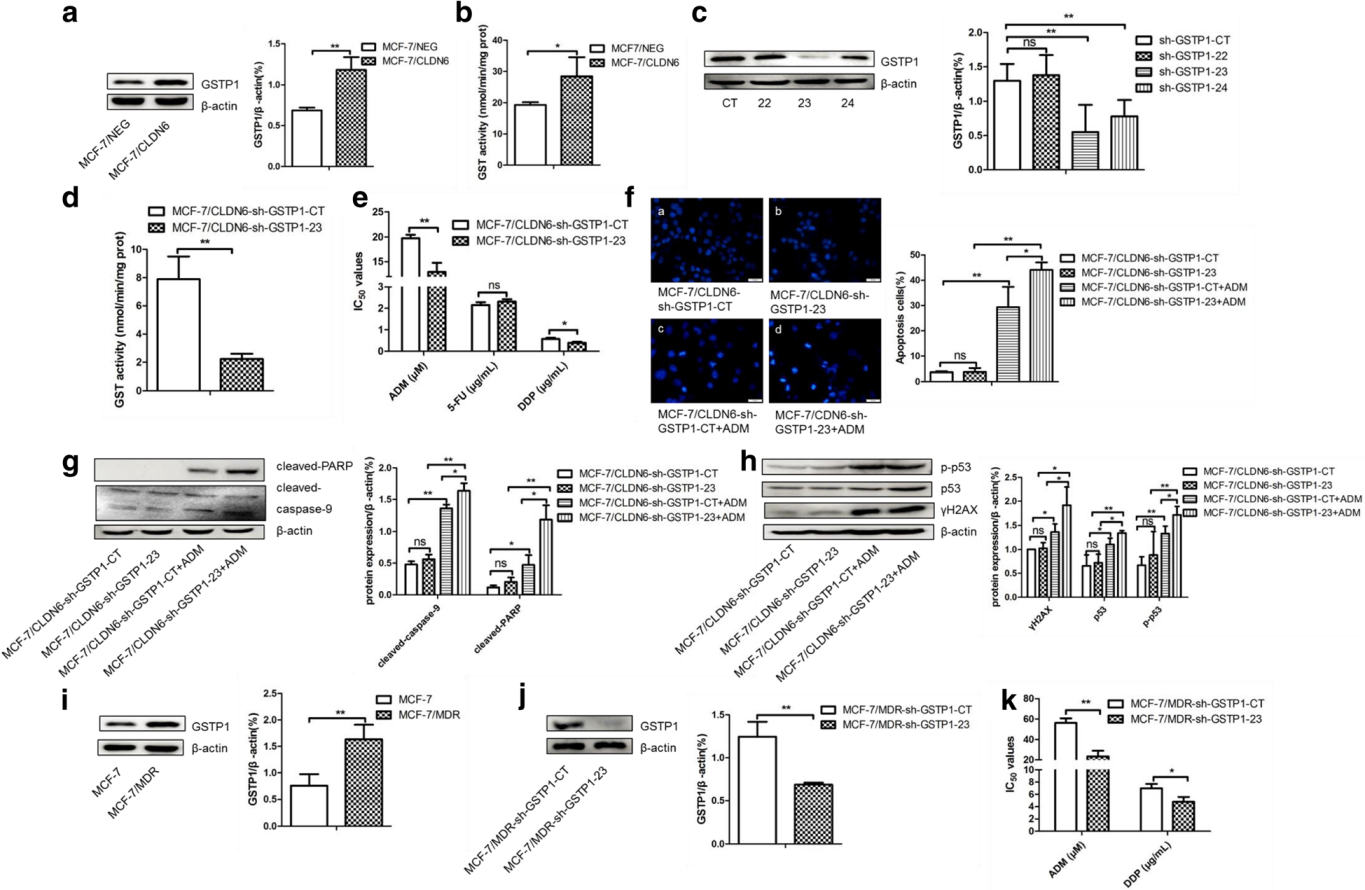
Silencing GSTP1 decreased chemoresistance in response to ADM in CLDN6-overexpressing MCF-7 cells. **a** GSTP1 expression was detected by western blot when CLDN6 overexpressed. **b** GST activity assay increased when overexpression CLDN6 in MCF-7 cells. **c** Three different GSTP1 specific shRNA constructs were utilized to screen the optimal sequence in MCF-7/CLDN6 cells by using western blot. **d** GST activity assay decreased when silenced GSTP1 expression in MCF-7/CLDN6 cells. **e** IC_50_ of ADM, DDP, and 5-FU when inhibited GSTP1 expression in CLDN6-overexpressing MCF-7 cells. **f** DAPI nuclear staining was applied to detect ADM-inducing apoptosis in MCF-7/CLDN6-sh-GSTP1 cells. **g** Cleaved-PARP, cleaved-caspase-9 and cleaved-caspase-7 expression in MCF-7/CLDN6-sh-GSTP1 treated with the same dose of ADM by using western blot assay. **h** Expression of γH2AX, p53, and p-p53 in GSTP1 silenced MCF-7/CLDN6 cells when treated with ADM. **i** GSTP1 expression was higher in MCF-7/MDR cells than in MCF-7 cells. **j** Silencing GSTP1 in MCF-7/MDR cells was verified by western blot. **k** IC_50_ of ADM and DDP when silenced GSTP1 expression in MCF-7/MDR cells. *,*P* < 0.05; **, *P* < 0.01

As a large numbers of studies confirmed its important role of GSTP1 in chemoresistance of breast cancer, our RNA-seq analysis also demonstrated upregulated the expression of GSTP1 in MCF-7/CLDN6 cells (Additional file 5: Figure S5), therefore we speculated that chemoresistance conferred by CLDN6 may be mediated by GSTP1 in these cells. Consistent with the above hypothesis, CLDN6 overexpression increased GSTP1 expression as well as GST enzyme activity as showed in Fig. 3a and b. Furthermore, multidrug resistance protein P-gp was also upregulated in MCF-7/CLDN6 cells (Additional file 6: Figure S6). To test whether GSTP1 is the key element in CLDN6 mediated chemoresistance, we constructed 3 distinct interference fragments of shRNA-GSTP1 plasmids and tested their functions. As shown in western blot analysis, sh-GSTP1–23 construct was the optimal interference fragment (Fig. 3c), transfection of sh-GSTP1–23 significantly silenced the expression of GSTP1 and decreased the GST enzyme activity simultaneously in MCF-7/CLDN6 cells (Fig. 3d). When the sh-GSTP1–23 plasmid was transferred into MCF-7/CLDN6 cells, IC_50_ of ADM and DDP decreased dramatically, from 19.74 ± 0.73 μM to 12.92 ± 1.86 μM and from 0.57 ± 0.06 μg/mL to 0.42 ± 0.05 μg/mL, respectively (Fig. 3e). Accordingly, ADM induced apoptosis and DNA damage increased significantly compared to controls (Fig. 3f–h). At the same time, we also verified elevated expression of GSTP1 in multidrug resistant breast cancer cells MCF-7/MDR as compared to MCF-7 cells (Fig. 3i). Silenced the expression of GSTP1 in MCF-7/MDR cells resulted in a significant decrease of IC_50_ for both ADM (from 56.11 ± 4.59 μM to 23.26 ± 5.68 μM) and DDP (from 6.97 ± 0.72 μg/mL to 4.77 ± 0.78 μg/mL) (Fig. 3j and k). These results confirmed that GSTP1 plays an important role in the acquisition of multidrug resistance in breast cancer cells.

**Fig. 4:**
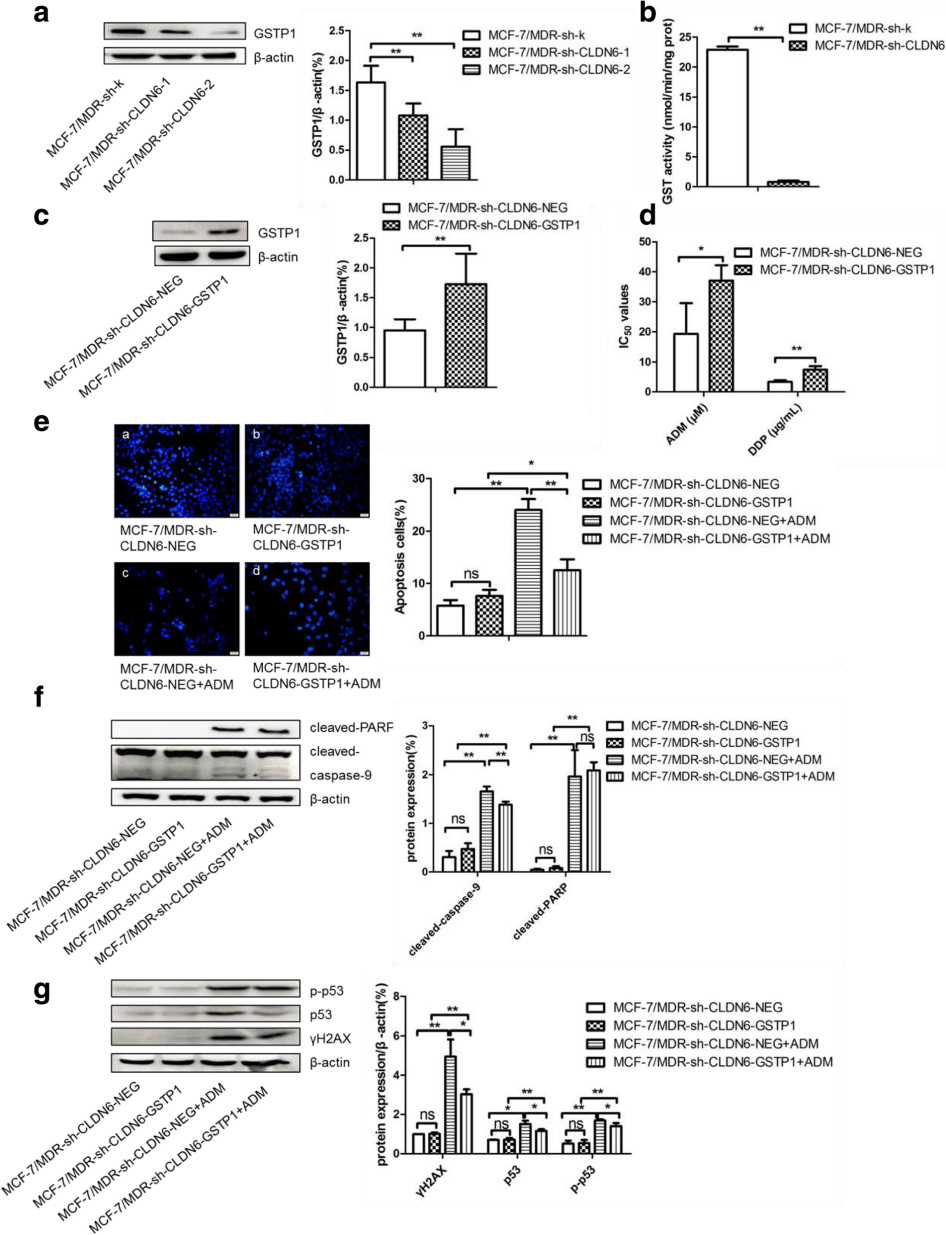
Overexpression of GSTP1 reversed CLDN6 silence-induced decreasing chemoresistance in MCF-7/MDR cells. **a** RNAi inhibited CLDN6 downregulated GSTP1 expression in MCF-7/MDR cells. **b** GST activity assay decreased when silenced CLDN6 in MCF-7/MDR cells. **c** Western blot verified GSTP1 overexpression in MCF-7/MDR-sh-CLDN6 cells. **d** IC_50_ of ADM and DDP when overexpressed GSTP1 in MCF-7/MDR-sh-CLDN6 cells. **e** DAPI nuclear staining was applied to detect ADM-inducing apoptosis when overexpressed GSTP1 in MCF-7/MDR-sh-CLDN6 cells. **f** Cleaved-PARP and cleaved-caspase-9 expression in GSTP1-overexpressed MCF-7/MDR-sh-CLDN6 cells treated with the same dose of ADM by using western blot assay. **g** Expression of γH2AX, p53, and p-p53 in GSTP1 overexpressed MCF-7/MDR-sh-CLDN6 cells when treated with ADM. *, *P* < 0.05; **, *P* < 0.01

Silenced the expression of CLDN6 in MCF-7/MDR cells reduced both GSTP1 expression and enzyme activity (Fig. 4a and b), whereas overexpression of GSTP1 in MCF-7/MDR-sh-CLDN6 cells (Fig. 4c) reversed their responses to ADM and DDP (Fig. 4d). Similarly, overexpression of GSTP1 reversed ADM induced apoptosis and DNA damage in these cells as tested by DAPI staining and western blot analysis (Fig. 4e–g). These results indicate that CLDN6 mediated breast cancer chemoresistance is through GSTP1.

### GSTP1 is regulated by p53 in CLDN6 overexpressing-MCF-7 cells

**Fig. 5:**
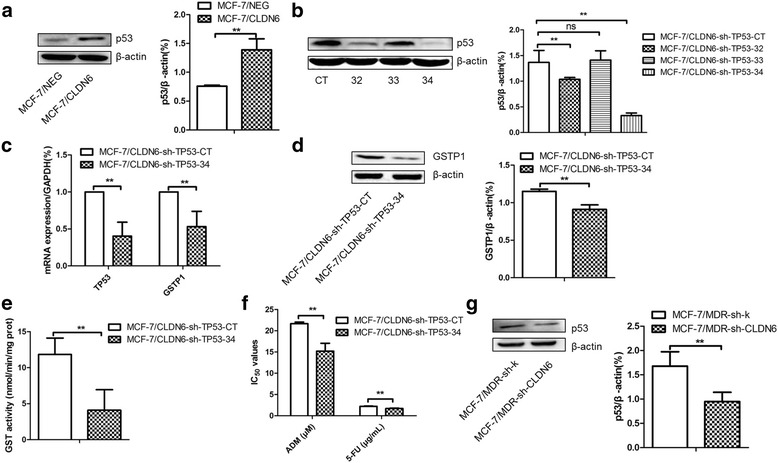
p53 regulated GSTP1 expression and chemoresistance in MCF-7 cells. **a** p53 expression when CLDN6 overexpression in MCF-7 cells was measured by western blot. **b** Three different TP53 specific shRNA constructs were utilized to screen the optimal sequence in MCF-7/CLDN6 cells by using western blot. **c** qPCR was applied the mRNA expression of GSTP1 when silenced TP53 gene in MCF-7/CLDN6 cells. **d** GSTP1 expression was observed when silenced TP53 gene by using western blot in MCF-7/CLDN6 cells. **e** GST activity was measured when inhibited TP53 gene expression. **f** IC_50_ of ADM and 5-FU when inhibited TP53 in MCF-7/CLDN6 cells. **g** Western blot analysis p53 expression in MCF-7/MDR cells when silenced CLDN6 gene. *, *P* < 0.05; **, *P* < 0.01

As CLDN6 mediates chemoresistance of MCF-7 and MCF-7/MDR cells through GSTP1, but how CLDN6 regulates the expression and activity of GSTP1 is still unclear. GSTP1 has been reported to be a novel transcriptional target of the p53 tumor suppressor gene, our western blot revealed that CLDN6 overexpression upregulated p53 expression in MCF-7 cells (Fig. 5a). Therefore we set up experiments to determine whether p53 regulates GSTP1 expression in breast cancer cells. Several shRNA-TP53 constructs were transfected into MCF-7/CLDN6 cells, optimal interference fragments were screened by western blot analysis (Fig. 5b). Inhibition of p53 expression in MCF-7/CLDN6 cells resulted significant decreases of GSTP1 in both mRNA and protein levels, as well as GST enzyme activity (Fig. 5c–e). Furthermore, the IC_50_ of ADM and 5-FU were also decreased from 21.68 ± 0.34 μM to 15.20 ± 1.86 μM and from 2.09 ± 0.14 μg/mL to 1.72 ± 0.04 μg/mL in these cells, respectively (Fig. 5f). Moreover, when CLDN6 expression was silenced by RNAi in MCF-7/MDR cells, expression of p53 was downregulated in accordance with GSTP1 (Fig. 5g).

### CLDN6 interacts with p53 and influences its subcellular localization

**Fig. 6:**
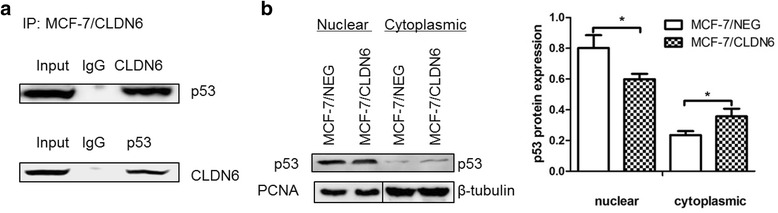
CLDN6 interacted with p53 and influenced p53 subcellular localization. **a** IP assay detected the interaction of CLDN6 and p53. **b** P53 protein expression in nuclear and cytoplasmic fractions of MCF-7/CLDN6 cells. PCNA was used as a control for nuclear protein and β-tubulin was used as a control for cytoplasmic protein. *,*P* < 0.05

The level of p53 protein was increased in MCF-7/CLDN6 cells. As CLDN6 is localized in the membranes of epithelial cells, how does CLDN6 influence p53 expression and function is the main point of our current experiment. IP results showed that CLDN6 interacted with p53 in MCF-7/CLDN6 cells (Fig. 6a). Nuclear and cytosol protein were extracted, and p53 distribution was examined. Our results demonstrated that nuclear p53 protein was decreased, whereas cytosol p53 protein was increased (Fig. 6b) in CLDN6 overexpressing MCF-7 cells. These results suggest that overexpression of CLDN6 results in redistribution of p53 protein in different cellular compartments.

### CLDN6 confers chemoresistance through GSTP1 to TNBC Hs578t cells

**Fig. 7:**
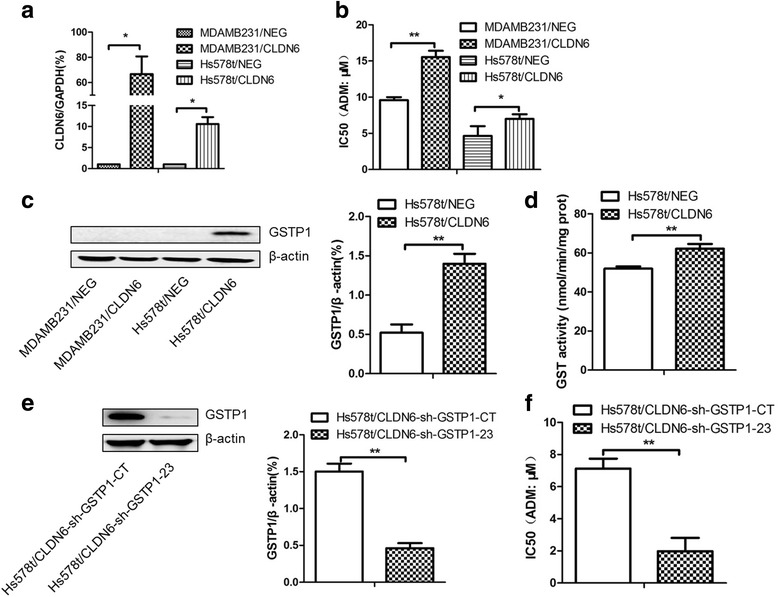
CLDN6 conferred chemoresistance through GSTP1 on TNBC Hs578t cells. **a** RT-qPCR verified CLDN6 overexpression in MDAMB231 and Hs578t cells. **b** IC_50_ of ADM when overexpressed CLDN6 in MDAMB231 and Hs578t cells. **c** GSTP1 expression when CLDN6 overexpressed in MDAMB231 and Hs578t cells. **d** GST activity was measured when CLDN6 overexpressed. **e** Silencing GSTP1 was verified by western blot in Hs578t/CLDN6 cells. **f** IC_50_ of ADM when silenced GSTP1 expression in Hs578t/CLDN6 cells. *,*P* < 0.05; **, *P* < 0.01

Triple negative breast cancers (TNBC) often have poor outcomes in clinics, and TNBCs are also claudin-low and cancer stem-like cells, currently no conventional chemotherapies can kill cancer stem cells. Overexpression of CLDN6 in two TNBC cell lines MDAMB231 and Hs578t increased ADM resistance (Fig. 7a and b). As the expression of GSTP1 was lost and TP53 gene was mutated in MDAMB231 cells, and CLDN6 expression up-regulated both GSTP1 expression and GST enzyme activity in Hs578t cells (Fig. 7c and d), we decided to investigate the role of GSTP1 in conferring chemoresistance to Hs578t cells only. We showed that silencing GSTP1 expression in Hs578t/CLDN6 cells resulted in a marked decreased IC_50_ of ADM from 7.13 ± 0.61 μM to 1.98 ± 0.84 μM (Fig. 7e and f), suggesting CLDN6 mediated chemoresistance through GSTP1 in TNBC Hs578t cells.

### Expression of GSTP1 is positively associated with CLDN6 in human breast cancers

**Fig. 8:**
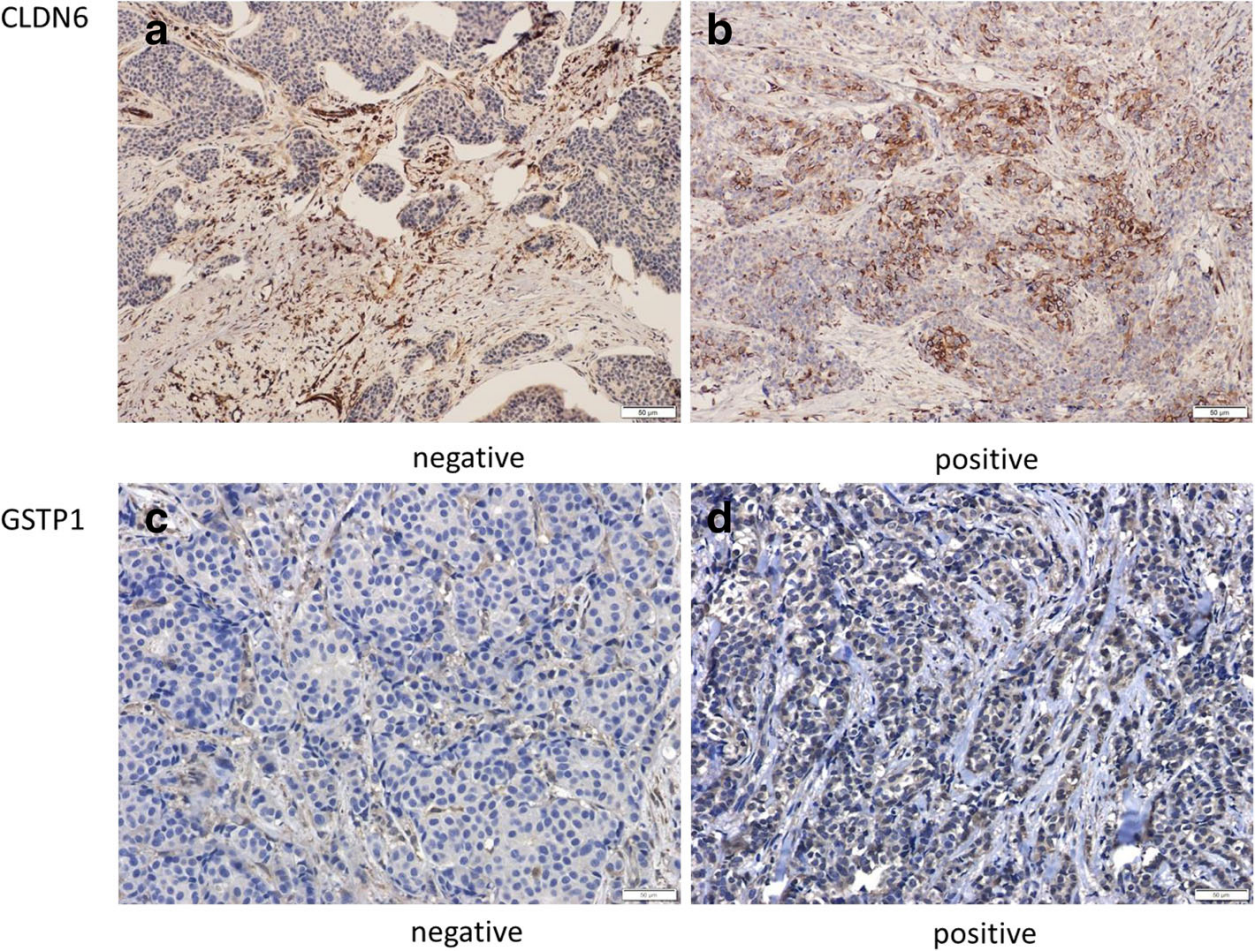
Expression of GSTP1 was associated with CLDN6 in human breast cancer samples. **a** and **b**. Immunohistochemistry revealed CLDN6 expression and distribution in breast cancer tissues. **c** and **d**. Immunohistochemistry revealed GSTP1 expression and distribution in breast cancer tissues

Finally, we analyzed the correlation between GSTP1 and CLDN6 expression in 40 human breast cancer tissues by using immunohistochemistry. The expression of CLDN6 mainly located in the membrane of breast cancer cells and GSTP1 was stained in the nuclear as showed in Fig. 8a-d. There is a strong positive correlation between the expressions of GSTP1 and CLDN6 (The Chi-square test, *P =* 0.02 < 0.05) (Table 1). The detailed results of the analysis are described in Additional file 7: Table S1. These data suggest that there is a CLDN6-GSTP1 regulatory axis in human breast cancer.

**Table 1:** Correlative analysis of CLDN6 and GSTP1 expression in breast cancer patients

CLDN6	GSTP1		
	–	+	++
–	2	7	1
+	11	9	4
++	0	1	3
+++	2	0	0
*P*	0.02 < 0.05		

## Discussion

Breast cancer is one of leading cause of premature death in developed and developing countries for women. Although significant advances have been achieved, chemoresistance remains a major clinical obstacle in successful treatment of breast cancer. Our pervious studies have confirmed that CLDN6 is significantly downregulated in breast invasive ductal carcinoma which correlates with lymphatic metastasis [3]. CLDN6 is therefore suggested to function as a tumor suppressor by inhibiting malignant phenotype of breast cancer cells through various signaling pathways such as p38-MAPK, JAKs-STATs, ASK1-JNK, and other pathways [4, 5]. As chemoresistance is a big problem facing the successful treatment of patients with breast cancer, and CLDN6 is expressed at a higher level in chemoresistant MCF-7/MDR cells as compared to parental MCF-7 cells, whether CLDN6 plays any role in conferring chemoresistance to breast cancer cells is unknown. In the current study, we explored the role of CLDN6 in breast cancer chemoresistance and the underlying mechanism, and found that high level expression of CLDN6 conferred chemoresistance on MCF-7 and MCF-7/MDR cells to ADM, 5-FU, and DDP through GSTP1. TNBCs are claudin-low breast cancers with the poorest prognosis [20]. We have also shown that overexpression of CLDN6 in TNBC cell line Hs578t increased ADM chemoresistance, accompanying with upregulated expression and enzyme activity of GSTP1. Inhibition of GSTP1 in Hs578t/CLDN6 cells decreased IC_50_ of ADM.

Previous studies have also showed that CLDN1, CLDN3, CLDN4, and CLDN7 expression are related with drug resistance to chemotherapy [8, 21–24]. CLDN3 and CLDN4, which are *Clostridium perfringens* enterotoxin (CPE) receptors, affected chemoresistance mediated by CPE in ovarian cancers [22, 23], it was also reported that CLDN3 and CLDN4 modulated the sensitivity to cisplatin partially through the copper and cisplatin influx transporter CTR1 [25]. CLDN7 increased chemosensitivity through the caspase pathway in human lung cancer cells [26]. One mechanism of CLDNs and chemoresistance acquisition may be related to cancer stem cells [27]. However, the mechanism of CLDN6 mediated chemoresistance is still unclear.

To explore the mechanism of CLDN6 mediated chemoresistance, using next generation sequencing techniques, we found that CLDN6-overexpressing MCF-7 cells (referred as MCF-7/CLDN6 cells) (Additional file 5: Figure S5) also expressed high level of GSTP1. Cytostatic drugs can be detoxified by enzymes, for example glutathione transferases, and GSTs can be induced during chemotherapy. Recent evidence indicated that GSTP1 directly involved in chemoresistance in multiple cancer cells [12, 13, 28, 29], we believed that GSTP1 may be an important gene in regulating chemoresistance in breast cancer MCF-7 cells. GSTP1 is often overexpressed in tumors and confer resistance against many types of drugs, including ADM and DDP. Similarly, a large numbers of studies also comfirmed the role of GSTP1 in chemoresistance in breast cancer [28, 30–32], overexpression of GSTP1 is linked to chemoresistance. Apart from P-gp, which is considered to be the most important multidrug resistance associated protein [33], is also overexpressed in the multidrug resistant cell line MCF-7/MDR. In consistence with our hypothesis, we found that, in addition to CLDN6 overexpression, GSTP1 expression and its enzyme activity were also increased in MCF-7/MDR cells as compared with the parental MCF-7 cells. Furthermore, silencing CLDN6 in MCF-7/MDR cells resulted in a decrease of both the expression and enzyme activity of GSTP1. To further demonstrated the cause relationship between CLDN6 and GSTP1, GSTP1 expression in MCF-7/CLDN6 cells was suppressed by siRNA (referred as MCF-7/CLDN6-sh-GSTP1–23 cells), both apoptosis and DNA damage were increased in MCF-7/CLDN6-sh-GSTP1–23 cells after ADM treatment, whereas overexpression of GSTP1 in MCF-7/MDR-sh-CLDN6 cells inhibited ADM induced apoptosis. These observations suggest that GSTP1 plays a crucial role in CLDN6 conferring chemoresistance on breast cancer MCF-7 cells.

In addition to glutathionylation and detoxification functions, GSTP1 has been shown to possess chaperone functions, regulation of nitric oxide pathways, and control over various kinases signaling pathways [34–37]. Furthermore, overexpression of GSTP1 is closely correlated with p53 in caner, and wtp53 positively regulates the expression of GSTP1 [38]. Accordingly, results presented in current study showed that the expression of p53 was positively correlated with GSTP1 in MCF-7 cells. Similarly, in cells with overexpressed CLDN6, p53 and GSTP1 were upregulated, shRNA inhibition of p53 expression significantly downregulated the expression of GSTP1 at both mRNA and the protein levels and its enzyme activity as well. In addition, inhibition of p53 also decreased the IC_50_ of ADM in MCF-7/CLDN6 cells. In accordance with above observations, silencing CLDN6 in MCF-7/MDR cells decreased both p53 and GSTP1 expressions. Therefore, upregulation of GSTP1 expression by CLDN6 in breast cancer MCF-7 cells is dependent upon p53. As a transmembrane protein, cytoplasmic tail of CLDN6 is the key domain responsible for their association with other binding protein [39]. Our results showed that cytosol p53 protein level was increased whereas nuclear p53 was decreased in cells with overexpressed CLDN6. IP experiment results showed CLDN6 interacted with p53 structurally, but how does CLDN6 interact with p53 still needs to be further studied.

## Conclusion

CLDN6-conferred chemoresistance on breast cancer is mediated by GSTP1, which is regulated by p53. CLDN6 structurally interacts with p53 protein and modulates its cellular distribution in human breast cancer cells. These results provide some clues on the mechanism of CLDN6 mediated chemoresistance and strategies to overcome chemoresistance in breast cancer.

## Additional files

Additional file 1: Figure S1. Flow cytometric analysis of cell apoptosis in CLDN6 knockdown MCF-7/MDR cells when treated with DDP. *, *P* < 0.05; **, *P* < 0.01. (PDF 435 kb)

Additional file 2: Figure S2. Flow cytometric analysis of cell cycle when knockdown CLDN6 in breast cancer multidrug resistance cell line MCF-7/MDR. *P* > 0.05. (PDF 55 kb)

Additional file 3: Figure S3. Flow cytometric analysis of cell apoptosis in CLDN6 overexpressed-MCF-7 cells when treated with DDP. (PDF 502 kb)

Additional file 4: Figure S4. Flow cytometric analysis of cell cycle in CLDN6 overexpressed-MCF-7 cells when treated with DDP. *P* > 0.05. (PDF 109 kb)

Additional file 5: Figure S5. Screened chemoresistance associated genes in RNA-seq analysis between MCF-7/CLDN6 and MCF-7/NEG cells. A. Genes associated with chemoresistance in RNA-seq were screened. B. qPCR was applied to verify genes expression. *P < 0.05; **P < 0.01. (PDF 80 kb)

Additional file 6: Figure S6. P-gp expression when CLDN6 overexpression in MCF-7 cells. **P < 0.01. (PDF 29 kb)

Additional file 7: Table S1. Clinicopathologic correlation between CLDN6 expression and clinicopathological factors of breast cancer tissues. (DOCX 16 kb)

## Abbreviations

5-FU: 5-fluorouracil
ADM: Adriamycin
cck8: Cell counting kit 8
CLDN6: Claudin-6
DAB: Diaminobenzidine
DAPI: 4′,6-diamidino-2-phenylindole
DDP: Cisplatin
FBS: Fetal bovine serum
GSTP1: Glutanthione S-transferase-p1
MDR: Multidrug resistance
PCR: Polymerase chain reaction
P-gp: P-glycoprotein
TNBC: Triple negative breast cancer
