# Supplementary figures and images for: CLDN6 promotes chemoresistance through GSTP1 in human breast cancer
Source: J Exp Clin Cancer Res. 2017 Nov 7;36:157. doi: 10.1186/s13046-017-0627-9 (PMC5678781; doi:10.1186/s13046-017-0627-9)

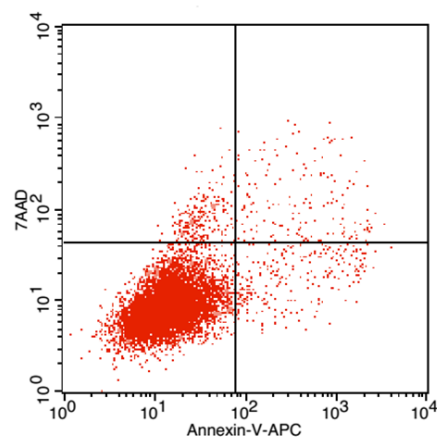

MCF-7/MDR-sh-k

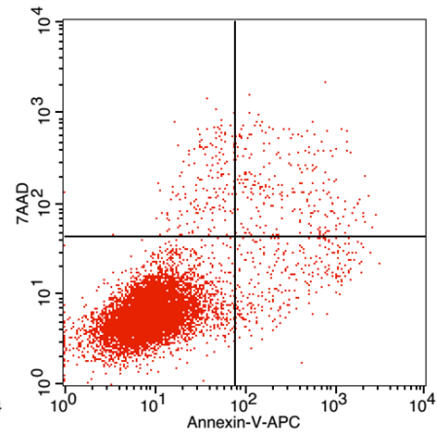

MCF-7/MDR-sh-CLDN6

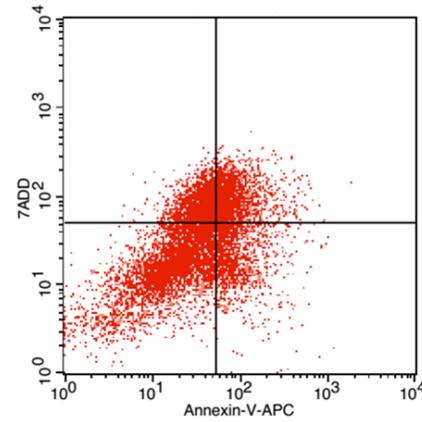

MCF-7/MDR-sh-k+DDP

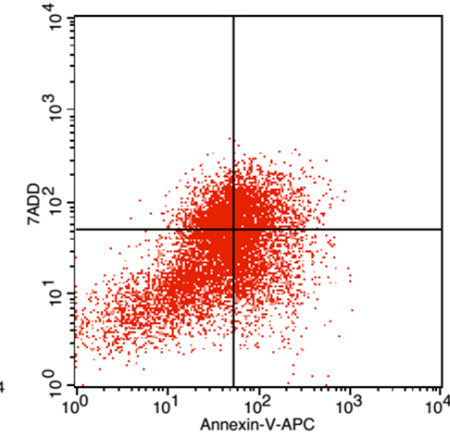

MCF-7/MDR-sh-CLDN6+DDP

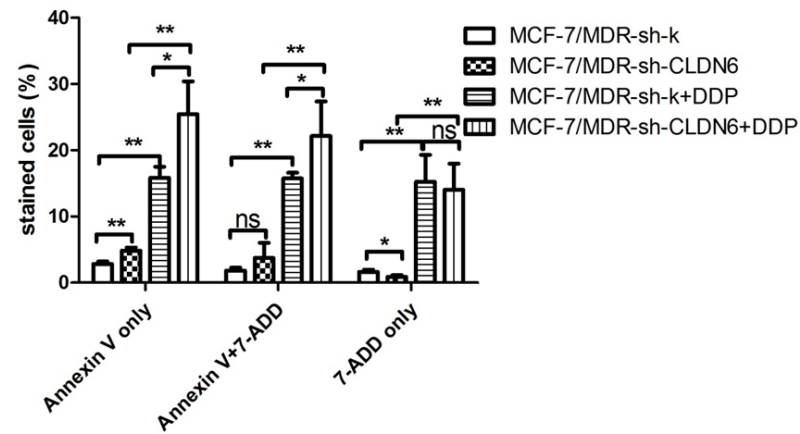

Supplement: Supplementary file 1 — Flow cytometric analysis of cell apoptosis in CLDN6 knockdown MCF-7/MDR cells when treated with DDP. *, P < 0.05; **, P < 0.01. (PDF 435 kb) [file 13046_2017_627_MOESM1_ESM.pdf]

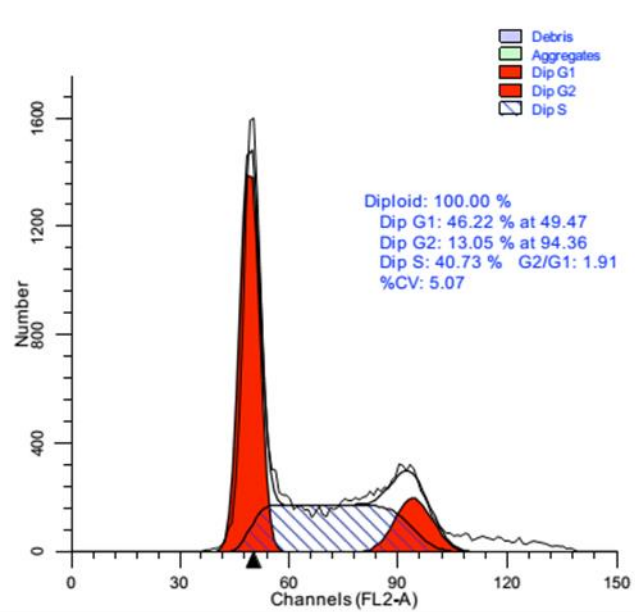

MCF-7/MDR-sh-k

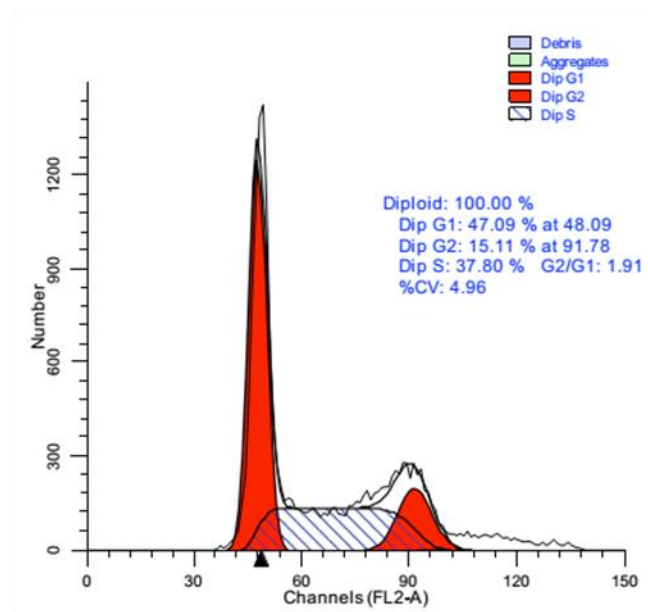

MCF-7/MDR-sh-CLDN6

Supplement: Supplementary file 2 — Flow cytometric analysis of cell cycle when knockdown CLDN6 in breast cancer multidrug resistance cell line MCF-7/MDR. P > 0.05. (PDF 55 kb) [file 13046_2017_627_MOESM2_ESM.pdf]

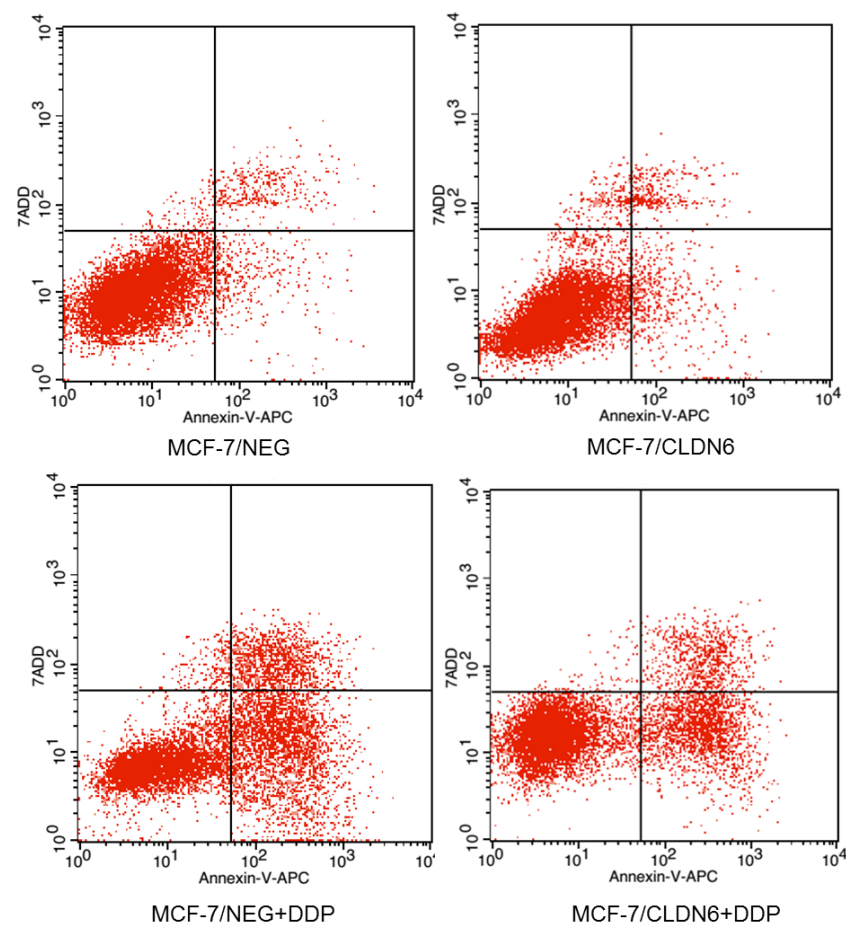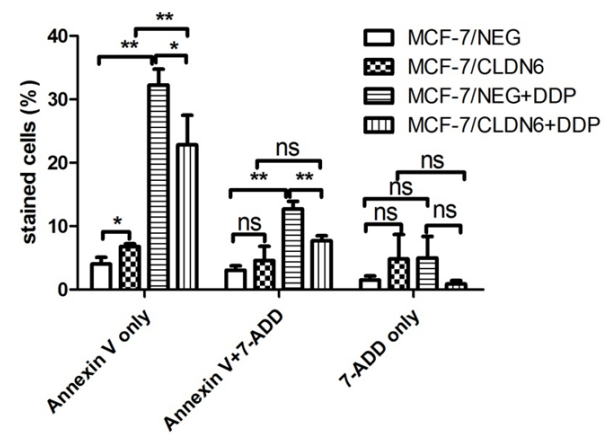

Supplement: Supplementary file 3 — Flow cytometric analysis of cell apoptosis in CLDN6 overexpressed-MCF-7 cells when treated with DDP. (PDF 502 kb) [file 13046_2017_627_MOESM3_ESM.pdf]

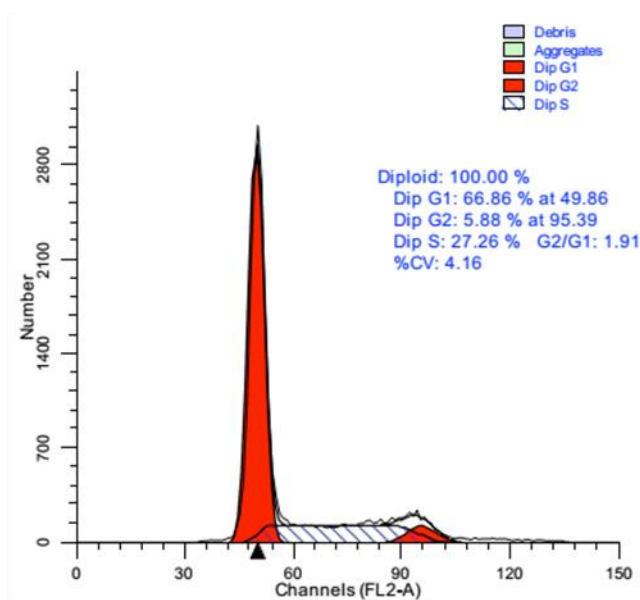

MCF-7/NEG

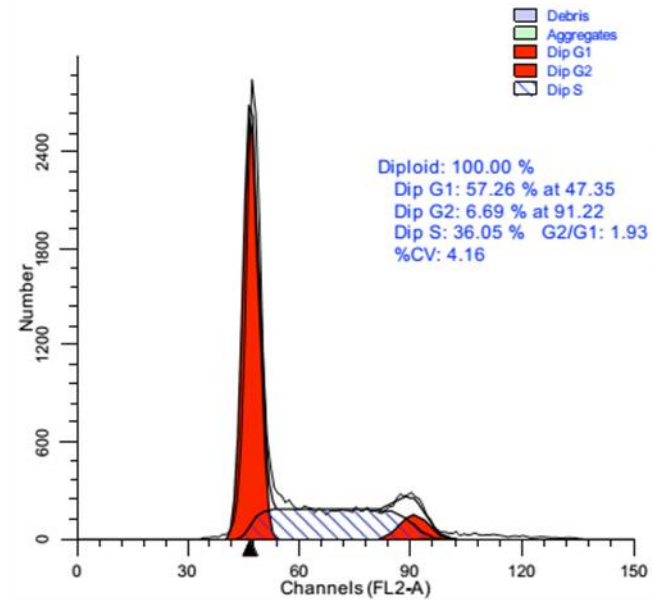

MCF-7/CLDN6

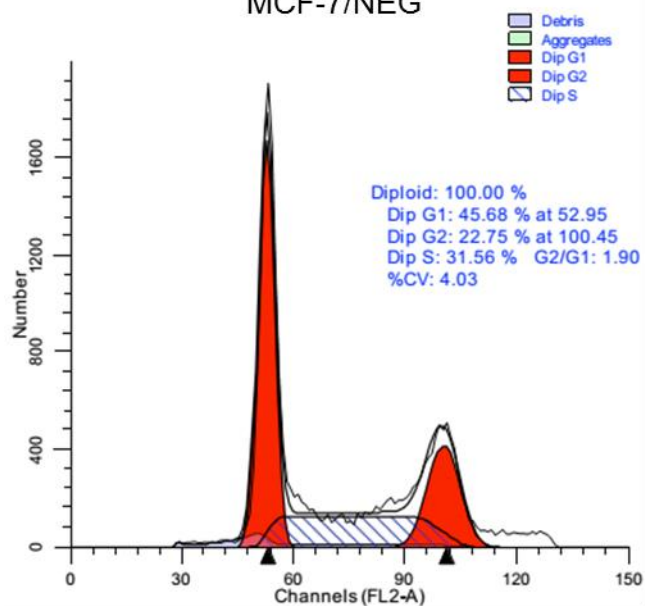

MCF-7/NEG+DDP

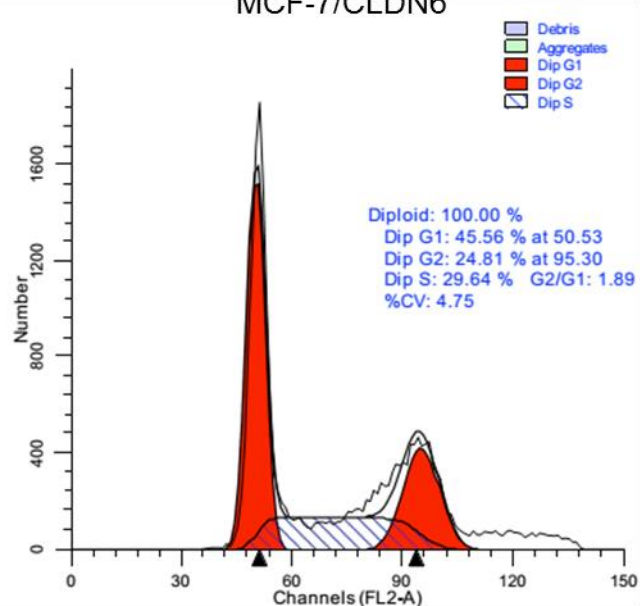

MCF-7/CLDN6+DDP

Supplement: Supplementary file 4 — Flow cytometric analysis of cell cycle in CLDN6 overexpressed-MCF-7 cells when treated with DDP. P > 0.05. (PDF 109 kb) [file 13046_2017_627_MOESM4_ESM.pdf]

A

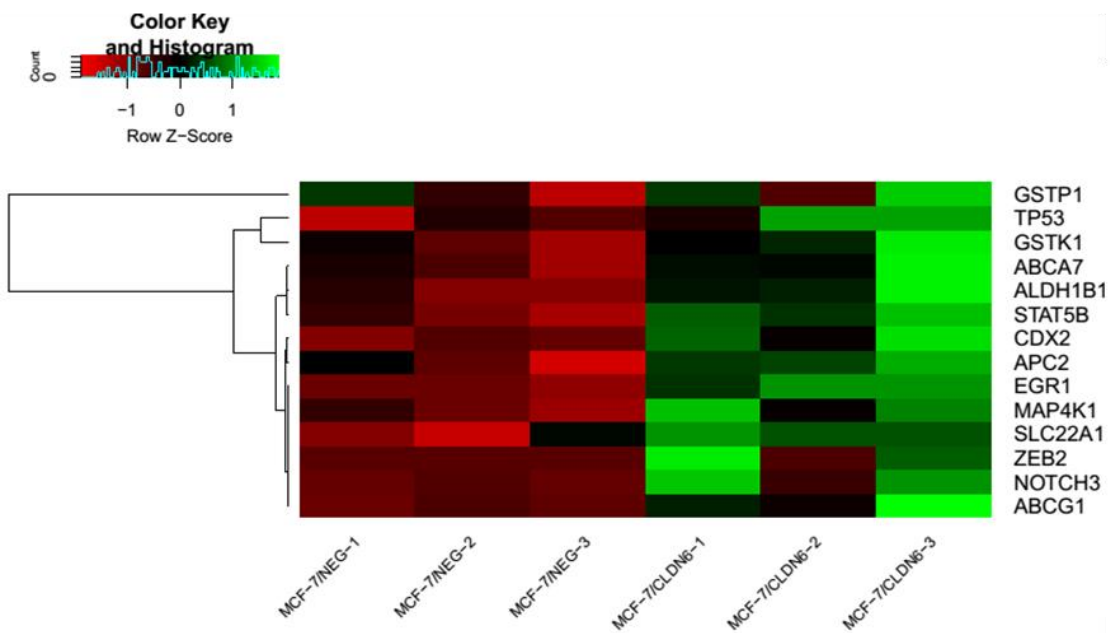

B

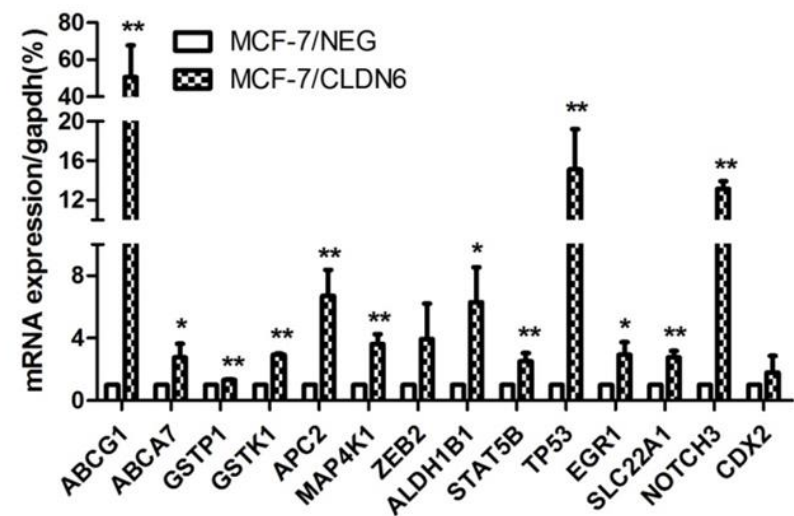

Supplement: Supplementary file 5 — Screened chemoresistance associated genes in RNA-seq analysis between MCF-7/CLDN6 and MCF-7/NEG cells. A. Genes associated with chemoresistance in RNA-seq were screened. B. qPCR was applied to verify genes expression. *P < 0.05; **P < 0.01. (PDF 80 kb) [file 13046_2017_627_MOESM5_ESM.pdf]

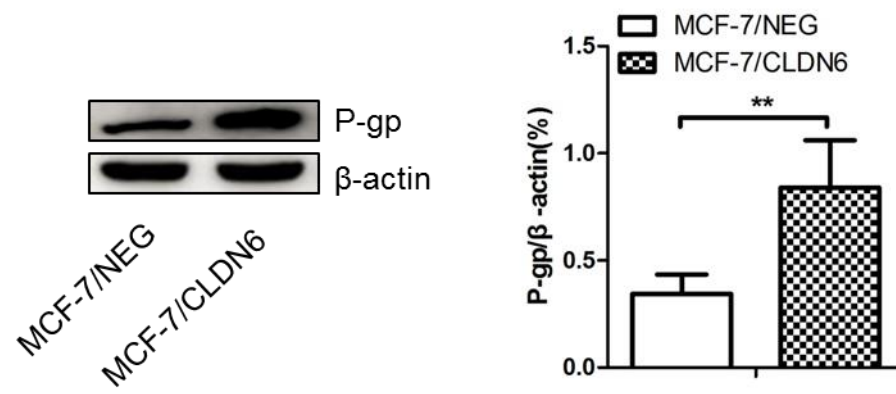

Supplement: Supplementary file 6 — P-gp expression when CLDN6 overexpression in MCF-7 cells. **P < 0.01. (PDF 29 kb) [file 13046_2017_627_MOESM6_ESM.pdf]
