# Supplementary material for: CLDN6 promotes chemoresistance through GSTP1 in human breast cancer
Source: J Exp Clin Cancer Res. 2017 Nov 7;36:157. doi: 10.1186/s13046-017-0627-9 (PMC5678781; doi:10.1186/s13046-017-0627-9)
Supplement: Supplementary file 7 — Clinicopathologic correlation between CLDN6 expression and clinicopathological factors of breast cancer tissues. (DOCX 16 kb) [file 13046_2017_627_MOESM7_ESM.docx]

Additional file 7: Table S1 Clinicopathologic correlation of CLDN6 expression in breast cancer patients

| Variables | CLDN6 | | | | | *P* |
| --- | --- | --- | --- | --- | --- | --- |
|  | - | + | ++ | | +++ |  |
| Age, years |  | | | | | |
| ≤40 | 0 | 2 | | 1 | 0 | 0.302 |
| 41-55 | 2 | 11 | | 0 | 1 |  |
| >55 | 8 | 11 | | 3 | 1 |  |
| Tumor size, cm |  | | | | | |
| ≤1 | 1 | 3 | | 1 | 0 | 0.801 |
| 1-2 | 7 | 12 | | 1 | 1 |  |
| >2 | 2 | 9 | | 2 | 1 |  |
| Lymph node metastasis |  | | | | | |
| <8 | 10 | 17 | | 4 | 2 | 0.130 |
| ≥8 |  | 7 | | 0 | 0 |  |
| Tumor (pTNM) stage |  | | | | | |
| Ⅰ | 0 | 1 | | 0 | 0 | 0.847 |
| Ⅱ | 5 | 11 | | 2 | 2 |  |
| Ⅲ | 5 | 7 | | 1 | 0 |  |
| Ⅳ | 0 | 0 | | 0 | 0 |  |
